# Annual acknowledgement of reviewers

**DOI:** 10.1186/1471-2393-13-28

**Published:** 2013-02-25

**Authors:** Emily Crow

**Affiliations:** 1 BioMed Central, 236 Grays Inn Rd, London, WC1X 8HB, United Kingdom

## Abstract

**Contributing reviewers:**

The editors of *BMC Pregnancy and Childbirth* would like to thank all our reviewers who have contributed to the journal in Volume 12 (2012).

## 

Edgardo Abalos

Argentina

Ishag Adam

Sudan

Kristina Adams Waldorf

USA

Richard Adanu

Ghana

Abdallah Adra

Lebanon

Ebenezer Afolabi

United Kingdom

A. J. Agopian

USA

Yenita Agus

Indonesia

Saifuddin Ahmed

USA

Stella Akinleye

Nigeria

Oluwarotimi Akinola

Nigeria

Mohammed Ali

Australia

Niloufer Ali

Pakistan

Gambo Aliyu

USA

Fernando Althabe

Argentina

Elizabeth Alvarez

Canada

Charles Ameh

United Kingdom

Nawal Ammar

Canada

Katie Anders

United Kingdom

Anne-Marie Nybo Andersen

Denmark

Lorraine Andrews

Ireland

Georgios Androutsopoulos

Greece

Diddy Antai

Nigeria

Laura Arbour

Canada

Megan Arbour

USA

Bronia Arnott

United Kingdom

Tengiz Asatiani

Georgia

Tigistu Adamu Ashengo

USA

Nathalie Auger

Canada

Ilona Autti-Rämö

Finland

Stella Babalola

USA

Regan Bailey

USA

Beth Bailey

USA

Patricia Bailey

USA

Rachel Bakker

Netherlands

Ileana Baldi

Italy

Mridula Bandyopadhyay

Australia

Ruben Barakat

Spain

Lesley Barclay

Australia

Janine Barden-O'Fallon

USA

Sarah Barnett

United Kingdom

Suzanne Barr

United Kingdom

Fernando Barros

Brazil

Robert Basaza

Uganda

Juliet Bedford

United Kingdom

Giorgio Bedogni

Italy

Katrien Beeckman

Belgium

Cecily Begley

Ireland

Mireille Bekker

Netherlands

Roberto Bellu

Italy

Marie Berg

Sweden

Thomas Bergholt

Denmark

João Bernardes

Portugal

Hind Beydoun

USA

Nita Bhandari

India

Sohinee Bhattacharya

United Kingdom

Zulfiqar Bhutta

Pakistan

Sibhatu Biadgilign

Ethiopia

Pauline Binder

Sweden

Colin Binns

Australia

Linda Birch

United Kingdom

Mary Anne Biro

Australia

Andrew Bisits

Australia

Maureen Black

USA

Andrew Blann

United Kingdom

Frank Bloomfield

New Zealand

Jennifer Blum

USA

Eric Blyth

United Kingdom

Lisa Bodnar

USA

Amy Bonomi

USA

Matthias Borchert

Germany

Carol Bower

Australia

Marek Brabec

Czech Republic

Amy Branum

USA

Leanne Bricker

United Kingdom

Anna Brytek-Matera

Poland

Sherri Bucher

USA

Anne Buist

Australia

Emmanuel Bujold

Canada

Jorge Burgos

Spain

Jessica Burke

USA

Joanne Cacciatore

USA

Leonie Callaway

Australia

Doris Campbell

United Kingdom

Michael Cannon

USA

Waldemar Carlo

USA

Mary Carolan

Australia

Giancarlo Castaman

Italy

Laura Caulfield

USA

Jose Cecatti

Brazil

Nils Chaillet

Canada

Tinnakorn Chaiworapongsa

USA

Beverly Chalmers

USA

James Chalmers

United Kingdom

Shiow-Ru Chang

Taiwan

Andrew Amos Channon

United Kingdom

Picklu Chaudhuri

India

Deepak Chawla

India

Jian Sheng Chen

Australia

Xinguang Chen

USA

Chie-Pein Chen

Taiwan

Yvonne Cheng

USA

Helen Cheyne

United Kingdom

Sunil Chhabra

India

See Kwee Ching

Malaysia

Saliya Chipwete

United Kingdom

Thanapan Choobun

Thailand

Mahbub Chowdhury

Bangladesh

John Cleland

United Kingdom

Ronald Cohen

USA

Erika Comasco

Sweden

Veerle Cossey

Belgium

Barbara Coughlan

Ireland

Debra Creedy

Australia

Jenny Cresswell

United Kingdom

Hannah Dahlen

Australia

Anne Kjersti Daltveit

Norway

Deirdre Daly

Ireland

Mary-Ann Davey

Australia

Matthias David

Germany

Deborah Davis

Australia

Ank De Jonge

Netherlands

Jan Willem De Leeuw

Netherlands

Patrizia De Marco

Italy

Adam De Smith

USA

Ilse Delbaere

Belgium

Thérèse Delvaux

Benin

Suzanne Demers

Canada

Fiona Denison

United Kingdom

Kebede Deribe

Ethiopia

Cate D'Este

Australia

Roland Devlieger

Belgium

Sulochana Dhakal

United Kingdom

Jodie Dodd

Australia

Utku Dogan

Turkey

France Donnay

USA

Signe Dørheim

Norway

Soo Downe

United Kingdom

Gerald Dubowitz

USA

Samuel Dumith

Brazil

Sada Nand Dwivedi

India

Christine East

Australia

Maigun Edhborg

Sweden

Solvig Ekblad

Sweden

Erling Ekerhovd

Sweden

Dawn Elder

New Zealand

Daniela Elleri

United Kingdom

David Ellwood

Australia

Janine Elson

United Kingdom

Offer Erez

Israel

Jan Jaap H.M. Erwich

Netherlands

David Eschenbach

USA

Jimmy Espinoza

USA

Hildah Essendi

United Kingdom

William Evans

USA

Daniel Exeter

New Zealand

Alex Ezeh

Kenya

Marcelo Farias

Chile

Antonio Farina

Italy

Diane Farrar

United Kingdom

Bukola Fawole

Nigeria

Marlena Fejzo

USA

Marilyn Felkner

USA

Xing Lin Feng

China

Jennifer Fenwick

Australia

John Ferguson

Australia

Christian Fiala

Austria

Fariyal Fikree

USA

Jane Fisher

Australia

Pierre Fournier

Canada

Karen Francis

Australia

Nicola Fratelli

Italy

Eva Haukeland Fredriksen

Norway

Atle Fretheim

Norway

Barbara Frohnert

USA

Elena Fuentes-Afflick

USA

Penny Furness

United Kingdom

Benjamin Gardner

United Kingdom

Deirdre Gartland

Australia

Asheber Gaym

Ethiopia

Alfredo Gei

USA

Ado Geidam

Nigeria

Ioannis Germanakis

Greece

Alison Gernand

USA

Labib Ghulmiyyah

Lebanon

Kristen Gibbons

Australia

Marij Gielen

Netherlands

Mika Gissler

Finland

Barbara Glare

Australia

Francois Goffinet

France

Katherine Gold

USA

Rachel Gold

USA

Audra Gollenberg

USA

Rebecca Gomperts

Netherlands

Adrienne Gordon

Australia

Francesca Gotsch

Italy

Jane Grassley

USA

Malcolm Griffiths

United Kingdom

Sorina Grisaru-Granovsky

Israel

Lars Grøvle

Norway

Kemal Güngördük

Turkey

Narcis Gusi

Spain

Atena Hafshejani

Australia

Hoda Harb

United Kingdom

Fiona Harris

United Kingdom

Yvonne Hauck

Australia

Geoff Hayes

USA

Alexander Heazell

United Kingdom

Renee Heffron

USA

Aura Heimonen

Finland

Peter Herbison

New Zealand

Bernardo Hernandez Prado

USA

Nicola Heslehurst

United Kingdom

Malene Hilden

Denmark

Emmet Hirsch

USA

Anders Hjern

Sweden

Nicolette Hodyl

Australia

Michael Holick

USA

Valerie Holmes

United Kingdom

Caroline Homer

Australia

Ayako Honda

South Africa

Monjurul Hoque

South Africa

Muhammad Hoque

South Africa

Shigeko Horiuchi

Japan

Rosemary Horne

Australia

Arash Hossein-Nezhad

Iran

Fon-Jou Hsieh

Taiwan

Carl Hubel

USA

Vanora Hundley

United Kingdom

Joke Aurelia Maria Hunfeld

Netherlands

Tai-Ho Hung

Taiwan

Jenny Hunt

Australia

Kristen Hurley

USA

Eileen Hutton

Canada

Anne-Mette Hvas

Denmark

Festus Ilako

Tanzania

Zubairu Iliyasu

Nigeria

Aamer Imdad

USA

Kazuo Itabashi

Japan

Debra Jackson

South Africa

Sig-Linda Jacobson

USA

Albrecht Jahn

Germany

Shohreh Jalaie

Iran

Rafat Jan

Pakistan

Patricia Janssen

Canada

Deborah Jones

USA

Laura Jones

United Kingdom

K Joseph

Canada

Davor Jurkovic

United Kingdom

Risto Kaaja

Finland

Francoise Malonga

Congo

S. M. Mostafa Kamal

Bangladesh

Joanne Katz

USA

Sarah Keim

USA

Marc Keirse

Australia

Tony Kelly

United Kingdom

Alan Kelly

Ireland

Kathleen Kendall-Tackett

USA

Charlotte Kenyon

United Kingdom

Joerg Kessler

Norway

Inaam Khalaf

Jordan

Asma Khalil

United Kingdom

Amina Khambalia

Australia

Iqbal Ansary Khan

Bangladesh

Young-Ho Khang

South Korea

Ali S Khashan

Ireland

Meharunnissa Khaskheli

Pakistan

Selina Khatun

Bangladesh

Hussein Lesio Kidanto

Tanzania

Tekoa King

USA

Carol Kingdon

United Kingdom

Ida Kirkegaard

Denmark

Torvid Kiserud

Norway

Michael Klein

Canada

Reija Klemetti

Finland

Rolf Klemm

USA

Marian Knight

United Kingdom

Saila Koivusalo

Finland

Paivi Kolu

Finland

Klaas Koop

Netherlands

Kristine Koski

Canada

Seni Kouanda

Burkina Faso

Michael Kramer

Canada

Su-Chen Kuo

Taiwan

Linda Kvist

Sweden

Anneke Kwee

Netherlands

Nicholas Lack

Germany

María Jesús Lagarda

Spain

Samantha Lain

Australia

Joan Lalor

Ireland

Helain Landy

USA

Taiwo Lawoyin

Nigeria

Anne Lee

USA

Robert Lindsay

United Kingdom

Gary Lipscomb

USA

Gustavo Lobato

Brazil

Marie Löf

Sweden

Natalia Lokhmatkina

United Kingdom

Carl Lombard

South Africa

Jody Lori

USA

Kei Lui

Australia

Zhong-Cheng Luo

Canada

Jennifer Lutomski

Ireland

Alison Macfarlane

United Kingdom

Elizabeth Machado

Brazil

Moke Magoma

Tanzania

Sarah Maheux-Lacroix

Canada

Syed Mahmood

India

Qun Mai

Australia

Abdulkarim Mairiga

Nigeria

Franz Majoko

United Kingdom

Ahmad Makuwani

United Kingdom

Mats Malqvist

Sweden

Albert Manasyan

Zambia

Joyce Marshall

United Kingdom

Susan Mason

USA

Matthews Mathai

Switzerland

Anne Matthews

Ireland

Andrew Mayers

United Kingdom

Gui Tarcisio Mazzoni

Brazil

Anthony Mbah

Nigeria

Fionnuala Mcauliffe

Ireland

Jennifer Mccall-Hosenfeld

USA

Affette Mccaw-Binns

Jamaica

Christine Mccourt

United Kingdom

David Mcdermott

USA

Alison Mcfadden

United Kingdom

Kevin Mcgeechan

Australia

Mark Mclean

Australia

Geraldine Mcneill

United Kingdom

Tarek Meguid

Malawi

Wubegzier Mekonnen

Ethiopia

Lotta Mellander

Sweden

Ramkumar Menon

USA

Fiona Mensah

United Kingdom

Maarit Mentula

Finland

Rebecca Merrill

USA

Sonja Merten

Switzerland

Laurie Meschke

USA

Marjo Metsäranta

Finland

Sayoki Mfinanga

Tanzania

Tomasz Miazgowski

Poland

Ornella Milanesi

Italy

Tracey Mills

United Kingdom

Karin Minnie

South Africa

Anne-Frederique Minsart

Belgium

Fadi Mirza

Lebanon

Ashna Mohangoo

Netherlands

Mohammed Mohsin

Australia

Alan Montgomery

United Kingdom

Maria Morales

Spain

Allisyn Moran

Bangladesh

John Morrison

Ireland

Laust Hvas Mortensen

Denmark

Michelle Mottola

Canada

Ellen Mozurkewich

USA

Rose Mpembeni

Tanzania

Mwifadhi Mrisho

Tanzania

Sia Msuya

Tanzania

Patrobas Mufubenga

Uganda

Stephen Munjanja

Zimbabwe

Deirdre Murphy

Ireland

Henry Murray

Australia

Victoria Nankabirwa

Uganda

Nadia Narchi

Brazil

John Newell

Ireland

Emanuele Nicastri

Italy

Susan Niermeyer

USA

Dorothea Nitsch

United Kingdom

Hedvig Nordeng

Norway

Abel Ntambue

Congo Democratic Republic

Stella Nyanzi

Uganda

Michael Obermeier

Germany

Colm O'Boyle

Ireland

Louise O'Brien

USA

Rhoune Ochako

Kenya

Clifford Odimegwu

South Africa

Pien M Offerhaus

Netherlands

Ellinor Olander

United Kingdom

Lemessa Oljira

Ethiopia

Pia Olsson

Sweden

Stephen Onwere

Nigeria

Peter O'Rourke

Australia

David Osrin

United Kingdom

Maha Othman

Canada

Kathryn Panaretto

Australia

Giancarlo Paradisi

Italy

Mary Parpinelli

Brazil

Angela Pascall

United Kingdom

Omrana Pasha

Pakistan

Megan Passey

Australia

Amar Patnaik

India

Luwei Pearson

Ethiopia

Rebecca Pearson

United Kingdom

Donald Peebles

United Kingdom

Andrea Barnabas Pembe

Tanzania

Lígia Peralta

Portugal

Franck Perrotin

France

Lars Plantin

Sweden

Katherine C Pollard

United Kingdom

Robert Powers

USA

Audrey Prost

United Kingdom

Liqian Qiu

China

Xingda Qu

Singapore

Zahida Qureshi

Kenya

Edwin Amalraj Raja

United Kingdom

Augustine Rajakumar

USA

Michael Ramharter

Gabon

Toril Rannestad

Norway

Shobha Rao

Indonesia

Chalapati Rao

Australia

Svein Rasmussen

Norway

Simmi Ratan

India

Anita Ravelli

Netherlands

Jo-Anne Rayner

Australia

Camille Raynes-Greenow

Australia

Pablo Rebollo

Spain

Tracy Reibel

Australia

Marvin Reid

Jamaica

Birgit Reime

Germany

Sylvia Reitmanova

Canada

Frida Renström

Sweden

Russel Rickard

USA

Marlies Rijnders

Netherlands

Christine Roberts

Australia

Devender Roberts

United Kingdom

Christopher Robinson

USA

Kristien Roelens

Belgium

Michael Ross

USA

Cristina Rossi

Italy

Rachel Rowe

United Kingdom

Kath Ryan

Australia

Padhraig Ryan

Ireland

Norkhafizah Saddki

Malaysia

Nandita Saikia

India

Sarah Saleem

Pakistan

Jason Salemi

USA

Kirsten Salmeen

USA

Jane Sandall

United Kingdom

Laura Sangaré

USA

Tuire Sannisto

Finland

Susan Santangelo

USA

Renske Schappin

Netherlands

Sicco Scherjon

Netherlands

Lone Schmidt

Denmark

Joann Schulte

USA

Kerry Schulze

USA

Erica Schytt

Sweden

Lai-Chu See

Taiwan

Omar Shaaban

Egypt

Sherri Shafer

USA

Prakeshkumar Shah

Canada

Antonia Shand

Australia

Monika Sharma

India

Allison Shorten

USA

Byera Shwekerela

Tanzania

Anna Maria Siega-Riz

USA

Bibha Simkhada

Nepal

Nigel Simpson

United Kingdom

Marlene Sinclair

United Kingdom

Prashant Kumar Singh

India

Paula Sisk

USA

Alkistis Skalkidou

Sweden

Nancy L Sloan

USA

Rhonda Small

Australia

Valerie Smith

Ireland

Debbie Smith

United Kingdom

Falko F Sniehotta

United Kingdom

Jonathan Snowden

USA

Tomas Sobotka

Austria

Hora Soltani

United Kingdom

Claudio Sosa

Uruguay

Eleazar Soto

USA

Ariani Souza

Brazil

Chandrashekhar Sreeramareddy

Nepal

Tomasina Stacey

United Kingdom

Anthony Staines

Ireland

Philip Steer

United Kingdom

Jelle Stekelenburg

Netherlands

Elizabeth Stenhouse

United Kingdom

Elizabeth Stenhouse

United Kingdom

Jonathan Stiles

USA

Sarah Stock

United Kingdom

Barbara Stoecker

USA

Katrine Strandberg-Laren

Denmark

Naeti Suksomboon

Thailand

Munira Sultana

Canada

Xiaojie Sun

China

Johanne Sundby

Norway

Gautham Suresh

USA

Geeta Swamy

USA

Miriam Taegtmeyer

United Kingdom

Tara Tancred

United Kingdom

David Tappin

United Kingdom

Charlotte Tawiah-Agyemang

Ghana

Minerva Thame

Jamaica

Nandita Thatte

USA

Rachel Thompson

USA

Gill Thomson

United Kingdom

Vijay Kumar Tiwari

India

Catherine Todd

USA

Elena Toffol

Finland

Van Tong

USA

Pat Tookey

United Kingdom

Maria Regina Torloni

Brazil

Andrea Tranquilli

Italy

Tomas Trnovec

Slovakia

Nazarius Mbona Tumwesigye

Uganda

Özge Tunçalp

USA

Raymond Tweheyo

Uganda

Niels Uldbjerg

Denmark

Regina Ungerer

Switzerland

Marcelo Urquia

Canada

Ihab Usta

Lebanon

Bettina Utz

United Kingdom

Bettina Utz

United Kingdom

Edi Vaisbuch

USA

Thomas Van Den Akker

Netherlands

Jossy Van Den Boogaard

Netherlands

Jacobus Van Der Velden

Netherlands

Elisabeth Van Leeuwen

Netherlands

Maria Van Pampus

Netherlands

Jos Van Roosmalen

Netherlands

Hans Van Rostenberghe

Malaysia

Giel Van Stralen

Netherlands

Edwin Van Teijlingen

United Kingdom

John Mg Van Vugt

Netherlands

Vicki Van Wagner

Canada

Stefanie Vandevijvere

Belgium

Siri Vangen

Norway

Paula Vargas Innocenti

Chile

Hayfaa Wahabi

Sudan

Peter Waiswa

Uganda

Lorraine Walker

USA

Carolina Walker

Spain

Stephen Walkinshaw

United Kingdom

L. Lewis Wall

USA

Denis Walsh

United Kingdom

Jane Warland

Australia

Andrew Weeks

United Kingdom

Alec Welsh

Australia

Daniel Westreich

USA

Heather Whitford

United Kingdom

Therese Wiegers

Netherlands

Carol Wilkins

United Kingdom

Andrew Willan

Canada

Jonathan Williams

United Kingdom

Amie Wilson

United Kingdom

Angie Wilson

United Kingdom

Tewes Wischmann

Germany

Ian Wright

Australia

Elizabeth Yakes

USA

Heather Yeatman

Australia

Lynn Yee

USA

Tegbar Yigzaw

Ethiopia

Yariv Yogev

Israel

Eivind Ystrom

Norway

Shumei Yun

USA

Jennifer Zeitlin

France

Abdhalah Ziraba

Kenya

## Pre-publication history

The pre-publication history for this paper can be accessed here:

http://www.biomedcentral.com/1471-2393/13/28/prepub

